# Pharmacokinetics Alterations in Critically Ill Pediatric Patients on Extracorporeal Membrane Oxygenation: A Systematic Review

**DOI:** 10.3389/fped.2020.00260

**Published:** 2020-06-26

**Authors:** Natalia Sutiman, Janine Cynthia Koh, Kevin Watt, Christoph Hornik, Beverly Murphy, Yoke Hwee Chan, Jan Hau Lee

**Affiliations:** ^1^Duke-NUS Medical School, Singapore, Singapore; ^2^Children's Intensive Care Unit, Department of Pediatrics, KK Women's and Children's Hospital, Singapore, Singapore; ^3^Department of Pediatrics, Duke University Medical Center, Durham, NC, United States; ^4^Duke Clinical Research Institute, Durham, NC, United States; ^5^Duke University Medical Center Library and Archives, Durham, NC, United States

**Keywords:** ECMO, extracorporeal, pharmacokinetics, disposition, pediatrics, critically ill

## Abstract

**Objectives:** This study aimed to identify alterations in pharmacokinetics in children on extracorporeal membrane oxygenation (ECMO), identify knowledge gaps, and inform future pharmacology studies.

**Data Sources:** We systematically searched the databases MEDLINE, CINAHL, and Embase from earliest publication until November 2018 using a controlled vocabulary and keywords related to “ECMO” and “pharmacokinetics,” “pharmacology,” “drug disposition,” “dosing,” and “pediatrics.”

**Study Selection:** Inclusion criteria were as follows: study population aged <18 years, supported on ECMO for any indications, received any medications while on ECMO, and reported pharmacokinetic data.

**Data Extraction:** Clearance and/or volume of distribution values were extracted from included studies.

**Data Synthesis:** Forty-one studies (total patients = 574) evaluating 23 drugs met the inclusion criteria. The most common drugs studied were antimicrobials (*n* = 13) and anticonvulsants (*n* = 3). Twenty-eight studies (68%) were conducted in children <1 year of age. Thirty-three studies (80%) were conducted without intra-study comparisons to non-ECMO controls. Increase in volume of distribution attributable to ECMO was demonstrated for nine (56%) drugs: cefotaxime, gentamicin, piperacillin/tazobactam, fluconazole, micafungin, levetiracetam, clonidine, midazolam, and sildenafil (range: 23–345% increase relative to non-ECMO controls), which may suggest the need for higher initial dosing. Decreased volume of distribution was reported for two drugs: acyclovir and ribavirin (50 and 69%, respectively). Decreased clearance was reported for gentamicin, ticarcillin/clavulanate, bumetanide, and ranitidine (range: 26–95% decrease relative to non-ECMO controls). Increased clearance was reported for caspofungin, micafungin, clonidine, midazolam, morphine, and sildenafil (range: 25–455% increase relative to non-ECMO controls).

**Conclusions:** There were substantial pharmacokinetic alterations in 70% of drugs studied in children on ECMO. However, studies evaluating pharmacokinetic changes of many drug classes and those that allow direct comparisons between ECMO and non-ECMO patients are still lacking. Systematic evaluations of pharmacokinetic alterations of drugs on ECMO that incorporate multidrug opportunistic trials, physiologically based pharmacokinetic modeling, and other methods are necessary for definitive dose recommendations.

**Trial Registration Prospero Identifier**: CRD42019114881.

## Introduction

Extracorporeal membrane oxygenation (ECMO) is a cardiopulmonary bypass support system used to temporarily sustain cardiac and/or respiratory function in critically ill patients. ECMO has been established as an effective modality in neonates and children who have failed conventional intensive care management. However, the ECMO circuit has been shown to sequester drug molecules in a highly unpredictable manner (1, 2) particularly with highly lipophilic and protein-bound drugs, as shown in a series of *ex vivo* experiments in which drugs were administered into isolated ECMO circuits (3, 4).

Pharmacokinetic studies conducted in critically ill neonates supported on ECMO have demonstrated marked alterations in drug pharmacokinetics and disposition. Specifically, the use of ECMO has been shown to increase the volume of distribution (*V*_*d*_) of drugs and alter the clearance of certain drugs (5, 6). Given the high variability in drug pharmacokinetics and disposition in patients on ECMO, a thorough understanding of the pharmacokinetic alterations that occur during ECMO is critical in guiding clinicians in determining dose adjustments.

To date, most studies involving pediatric ECMO clinical pharmacology have been conducted with anti-infective agents, but data have started to emerge for other classes of commonly used drugs in this patient population. However, there is currently no systematic review of the current evidence base of the alterations in drug pharmacokinetics and disposition in children supported on ECMO. We therefore performed this systematic review to assess and summarize the current literature up for alterations in drug pharmacokinetics, specifically clearance and *V*_*d*_, in critically ill pediatric patients supported on ECMO.

## Materials and Methods

This systematic review was registered in PROSPERO with the registration number CRD42019114881.

### Search Strategy

A systematic, computerized search of the literature in MEDLINE (via PubMed), CINAHL, and Embase was conducted by a medical research librarian (B.M.) using a controlled vocabulary and keywords related to *extracorporeal membrane oxygenation* (ECMO) and *pharmacokinetics, pharmacology, drug disposition*, or *dosing* in the *pediatric* population (from birth to 18 years). Our search time frame was from database inception to November 1, 2018. The search strategies are shown in the Supplementary Information 1. The reference lists of all selected publications were checked to retrieve relevant publications that were not identified in the computerized search.

### Study Selection and Risk-of-Bias Assessment

The final search results were compiled and imported into Covidence (Veritas Health Innovation, Melbourne, Australia). Two reviewers (N.S. and J.K.) independently screened and reviewed titles and abstracts to assess their eligibility. Full-text articles were retrieved if the abstract provided insufficient information to establish eligibility or if the article passed the first eligibility screening. Disagreements on study eligibility were resolved by consensus or by arbitration from a third independent reviewer (J.H.L).

Studies that fulfilled the following inclusion criteria were included: (1) study population aged 18 years and below, including neonates (0–28 days of age), infants (29 days−1 year of age), children (>1–12 years of age), and adolescents (>12–18 years of age), (2) study population receiving ECMO for any clinical indications and durations, (3) study population receiving any medications or therapeutics while on ECMO, and (4) studies reporting pharmacokinetic parameters, specifically clearance and *V*_*d*_, in patients supported on ECMO. Case reports, case series, abstracts, and conference proceedings were all included. We only included studies published in the English language. Articles with only pharmacodynamics, response, or safety data were not included. Other exclusion criteria included (1) *ex vivo* studies, (2) animal studies, and (3) studies with predominantly adult population with no separate description of a pediatric subgroup. Given the nature of pharmacokinetic studies, traditional risk-of-bias assessment with tools, such as the Newcastle–Ottawa scale, was not conducted. Instead, each article was evaluated for completeness of reporting based on the consensus-based ClinPK statement by Kanji et al. (7).

### Data Extraction and Synthesis

A standardized data collection form was used to extract the relevant data from each eligible study. The following data were collected: key characteristics of the study (e.g., study year, study design, and type of publication), characteristics of the study population (e.g., age, clinical indications of ECMO, and modality of ECMO), interventions received by the study population (e.g., medications received while on ECMO, including their dosage form and dose regimen), and pharmacokinetic parameters measured or estimated. For the purpose of this review, we specifically focused on two pharmacokinetic parameters—clearance and *V*_*d*_.

### Comparison of Clearance and Volume of Distribution of Drugs Between the ECMO and Non-ECMO Groups

We extracted clearance and/or *V*_*d*_ values from the included studies and compared these pharmacokinetic parameters between children supported with ECMO and children not on ECMO. For studies with intra-study comparators, the pharmacokinetic parameters were compared between the ECMO and non-ECMO groups within each study. For studies that did not include non-ECMO control groups, we compared these pharmacokinetic parameters against historical controls in other pharmacokinetic studies conducted in pediatric patients not supported with ECMO. For each drug, a computerized search was conducted in MEDLINE (via PubMed) using keywords related to *the drug* and *pharmacokinetics, pharmacology*, or *dosing* in the *pediatric* population (from birth to 18 years). The titles and abstracts of the articles were screened, and full-text articles were subsequently reviewed for pharmacokinetic parameters of the drug and its metabolites (where applicable) in non-ECMO pediatric patients. Wherever possible, the non-ECMO historical controls were age-matched against the ECMO group. In addition, to evaluate the association between changes in *V*_*d*_ or drug clearance and the drug's physicochemical properties, we compared the log *P*-values between drugs with increased, decreased, or no change in *V*_*d*_ or drug clearance.

### Assessment of Quality of Reporting of Pharmacokinetic Studies

We evaluated each study included in this systematic review for their compliance rates with each item in the ClinPK checklist, a 24-item checklist for transparent and consistent reporting of clinical pharmacokinetic studies developed by Kanji et al. (7). The checklist included criteria such as study rationale, eligibility criteria of study participants, coadministration of study drugs with potentially interacting drugs, validation of quantitative bioanalytical methods, pharmacokinetic modeling methods, and reporting of results with appropriate measures.

## Results

### Characteristics of Included Studies and Study Populations

**Figure 1:**
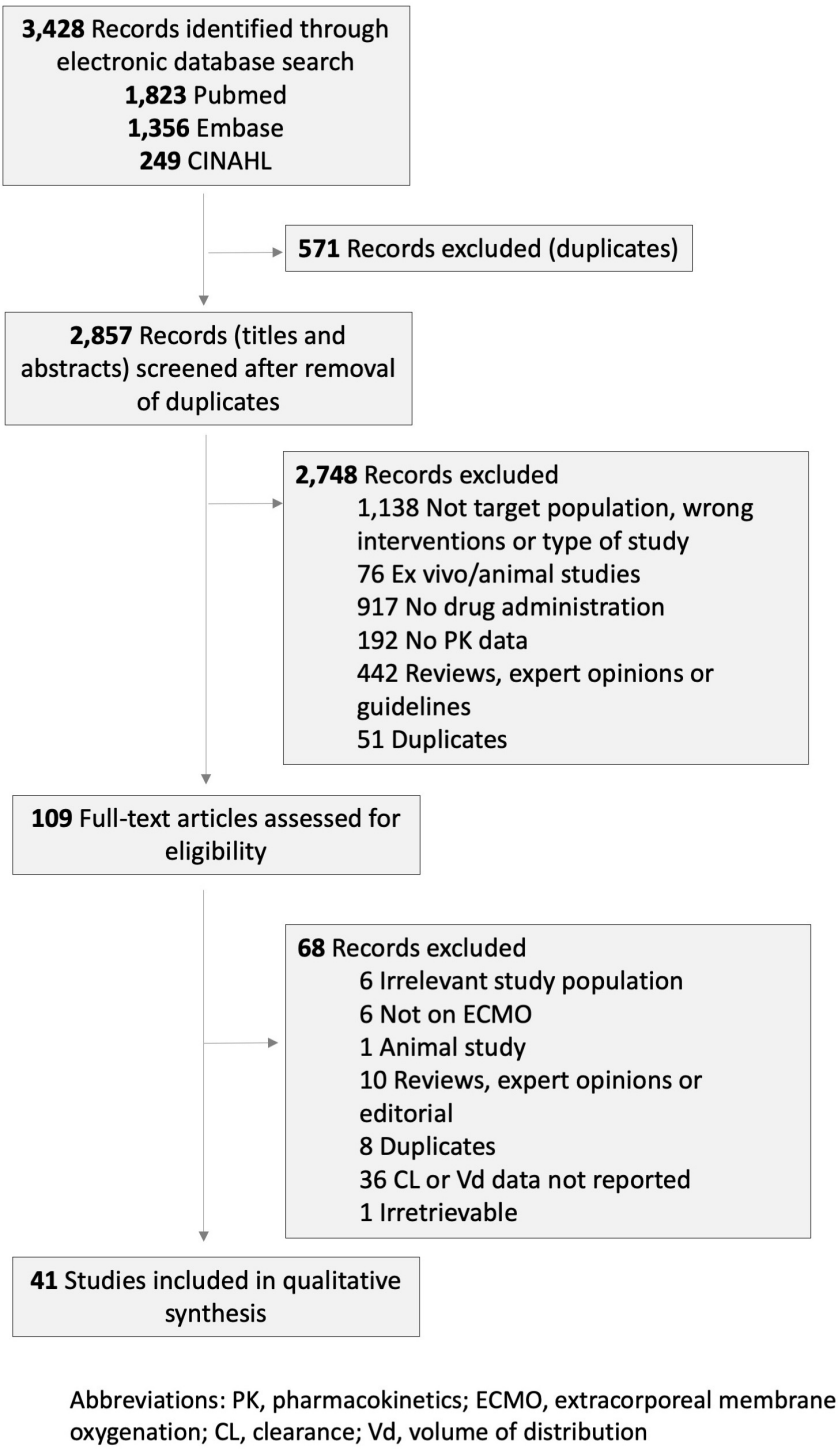
PRISMA diagram of study selection.

Out of the 3,428 records retrieved by the systematic search and hand search of reference lists, 41 studies met the inclusion criteria and included a total of 574 pediatric patients (Figure 1). These 41 publications reported clearance and *V*_*d*_ data for 23 drugs: six antibiotics (cefotaxime, gentamicin, meropenem, piperacillin/tazobactam, ticarcillin/clavulanate, and vancomycin), three antiviral agents (acyclovir, oseltamivir, and ribavirin), four antifungal agents (caspofungin, fluconazole, micafungin, and voriconazole), three anticonvulsants (fosphenytoin, levetiracetam, and phenobarbital), and others (bumetanide, clonidine, heparin, midazolam, morphine, ranitidine, and sildenafil). Twenty-eight studies (68%) were conducted in children <1 year of age. The clinical and methodological characteristics of included studies are summarized in Table 1 and Supplementary Table 1. The studies were published between 1989 and 2018.

**Table 1:** Characteristics of included studies and study populations.

**Drug**	**Total no. of patients, *N***	**Study population*^a^***	**Age, median (IQR) [range], days**
**Antibiotics**			
Cefotaxime (8)	37	Neonates and infants	[1–199]
Gentamicin (9–14)	120	<18 years	[2–399]
Meropenem (15–17)	3	<18 years	[10–1,460]
Piperacillin/tazobactam (18)	6	Neonates and infants	[8–210]
Ticarcillin/clavulanate (19)	2	Adolescents	[2,190–2,372.5]
Vancomycin (20–25)	188	<18 years	[0–5,510]
**Antivirals**			
Acyclovir (26)	1	Infant	Not specified
Oseltamivir (27)	1	Adolescent	2,190
Ribavirin (28)	1	Neonate	14
**Antifungals**			
Caspofungin (29)	1	Infant	330
Fluconazole (30, 31)	31	<18 years	[1–6,498]
Micafungin (32)	12	<18 years	[0–574]
Voriconazole (33, 34)	2	<18 years	3,056 [1,825–6,205]
**Anticonvulsants**			
Fosphenytoin (35)	6	<18 years	Not specified
Levetiracetam (36)	1	Adolescent	5,840
Phenobarbital (35, 37, 38)	28	<18 years	Not specified
**Others**			
Bumetanide (39)	11	Neonates	[1–7]
Clonidine (40)	22	Infants	30 (192)
Heparin (41)	12	<18 years	[0–5,910]
Midazolam (42, 43)	40	Neonates	[0.17–18]
Morphine (44, 45)	25	Neonates	[0–9]
Ranitidine (46)	13	Neonates	[0–4]
Sildenafil (47)	11	Neonates and infants	20 [2–121]

a *Neonates: 0–28 days of age, infants: 29 days−1 year of age, children: >1–12 years of age, and adolescents: >12–18 years of age*.

There was substantial heterogeneity in the pharmacokinetic sampling and treatment modalities used across studies. These variations included differences in route of administration (intravenous or oral), dosages of medications, administration method (bolus, intermittent dosing, or continuous infusion), and usage of co-medications. Twelve of the 41 studies (29.3%) included patients on continuous renal replacement therapy (CRRT) (15, 16, 19, 20, 26–28, 30, 31, 33, 35, 42). Renal and liver functions were not consistently reported in majority of the studies. Specifically, baseline renal functions were reported only in 19 out of 41 studies (46.3%) (8–11, 20–24, 28, 30–33, 37, 39, 40, 43, 46) while only 12 out of 41 studies (29.3%) reported baseline liver functions (8, 10, 21, 24, 28, 30–32, 40, 42, 43, 46). In addition, 17 studies (41%) did not report the type of ECMO used (11–13, 16–19, 22, 25–27, 30, 31, 38, 41, 44, 47). Of those that reported the type of ECMO used, 10 studies (24%) employed both veno-venous (VV) and venous–arterial (VA) ECMO (8, 14, 20, 24, 32, 35, 40, 42) 10 studies (24%) used VA ECMO only (9, 15, 21, 23, 28, 37, 39, 43, 45, 46), and the remaining four studies (10%) used VV ECMO only (29, 33, 34, 36). Of 41 studies, 21 studies (51.2%) specified the type of oxygenator used, with Avecor 0800 oxygenator (Avecor Cardiovascular Inc.) being the most commonly used (8, 10, 15, 17, 21–24, 28, 31, 34, 36–38, 40, 42–46, 48). However, only 14 studies (34.1%) reported the prime volume (range: 200–750 ml) (8, 11, 14, 15, 21, 24, 31, 32, 37, 40, 43, 44, 48, 49). Only seven studies (17%) were conducted with intra-study comparisons to non-ECMO controls (9, 12, 22, 30, 34, 35, 40).

### Alterations in Volume of Distribution of Drugs in ECMO-Supported Patients

Of the 23 drugs studied, *V*_*d*_ values were reported for 16 drugs (70%) (8–14, 18, 20–24, 26, 28, 30–40, 42, 43, 47, 50) with the remaining seven other drugs having only clearance values reported without *V*_*d*_ values (15–17, 19, 27, 29, 44–46). Relative to non-ECMO controls, significant alterations in *V*_*d*_ were observed in pediatric patients supported on ECMO for 11 of 16 studied drugs (Table 2). Increased *V*_*d*_ attributable to ECMO was demonstrated for nine drugs: cefotaxime, gentamicin, piperacillin/tazobactam, fluconazole, micafungin, levetiracetam, clonidine, midazolam, and sildenafil (range: 23.8–345% increase relative to non-ECMO controls), suggesting the need for higher initial dosing (11, 16, 17, 22, 32, 39, 41, 46, 47). Decreased *V*_*d*_ was reported for two drugs: acyclovir and ribavirin (50 and 69%, respectively) (26, 28). *V*_*d*_ of the following drugs were not substantially altered in patients supported on ECMO relative to non-ECMO controls: vancomycin, voriconazole, fosphenytoin, phenobarbital, and bumetanide (2, 22, 35, 39).

**Table 2:** Summary of pharmacokinetic changes (*V*_*d*_ and clearance) published in critically ill pediatric patients on ECMO.

**Drug**	**Volume of distribution (% change)**	**Clearance (% change)**
**Antibiotics**		
Cefotaxime (8, 51, 52)	↑*^f^* (23.8)	↔*^f^*
Gentamicin (9–14)	↑*^a^* (28.8–58.8)	↓*^a^* (26.3–31.7)
Meropenem (15–17, 49, 53)	NA	↔*^f^*
Piperacillin/tazobactam (18, 54)	↑*^f^* (37.3)	NA*^d^*
Ticarcillin/clavulanate (19, 55)	NA*^c^*	↓*^f^* (46.8)
Vancomycin (20–25)	↔^a^^,^^b^	↔^a^^,^^b^
**Antivirals**		
Acyclovir (26, 56)	↓*^f^* (50)	NA*^d^*
Oseltamivir (27, 57)	NA*^c^*	↔*^f^*
Ribavirin (28, 58, 59)	↓*^f^* (69.3–82.2)	NA*^d^*
**Antifungals**		
Caspofungin (29, 60)	NA*^c^*	↑*^f^* (455)
Fluconazole (30, 31)	↑*^a^* (39.8)	↔*^a^*
Micafungin (32, 61)	↑*^f^* (80.8)	↑*^f^* (105)
Voriconazole (33, 34)	↔*^a^*	↔*^a^*
**Anticonvulsants**		
Fosphenytoin (35)	↔*^a^*	NA*^d^*
Levetiracetam (36, 62, 63)	↑*^f^* (33.6–242)	↔
Phenobarbital (35, 37, 38)	↔*^a^*	NA*^d^*
**Others**		
Bumetanide (39, 64, 65)	↔*^f^*	↓*^f^* (42.7–83.8)
Clonidine (40)	↑*^a^* (55)	↑*^a^* (100)
Heparin (41)	NA*^e^*	NA*^e^*
Midazolam (42, 43, 66, 67)	↑*^f^* (188–345)	↑*^f^* (192)
Morphine (44, 45)	NA*^c^*	↑*^b^* (84.3)
Ranitidine (46, 68)	NA*^c^*	↓*^f^* (94.7)
Sildenafil (47, 69)	↑*^f^* (68.9)	↑*^f^* (24.7)

*NA, not available; ↑, increased relative to non-ECMO controls; ↓, decreased relative to non-ECMO controls; ↔, equal to non-ECMO controls*.

a *Clearance and V_d_ values between ECMO and non-ECMO groups were compared within the same studies*.

b *Clearance and V_d_ have been shown to be altered compared to the non-ECMO group when comparisons are made with historical controls*.

c *Not available because volume of distribution was not measured and reported*.

d *Not available because clearance was not measured and reported*.

e *Not available because data on non-ECMO comparators were not available*.

f *Clearance and V_d_ values between ECMO and non-ECMO groups were compared against historical controls*.

Of note, vancomycin was studied in six separate studies, only one of which included a non-ECMO comparator group (22) while the remaining five did not (20, 21, 23, 24, 50) Although a trend was observed toward smaller *V*_*d*_ of vancomycin relative to non-ECMO controls, the difference did not reach statistical significance in the study by Buck (22) However, when the *V*_*d*_ values were compared against historical controls, increased *V*_*d*_ was observed compared to pediatric patients not on ECMO (20, 21, 23, 24, 50). In addition, we did not identify any significant correlation between lipophilicity (as measured by log *P*) of drugs and changes in *V*_*d*_ attributable to ECMO (Supplementary Table 1).

### Alterations in Drug Clearance in ECMO-Supported Patients

Out of the 23 drugs studied, drug clearance was reported for 17 drugs (74%) (8–17, 19–24, 27, 29–32, 36, 39, 40, 42–47, 50) with the remaining five other drugs having only *V*_*d*_ values reported (18, 26, 28, 35, 37, 38). Compared to non-ECMO controls, significant changes in clearance were observed in pediatric patients supported on ECMO for 10 of 17 studied drugs (Table 2). Decreased clearance was reported for gentamicin, ticarcillin/clavulanate, bumetanide, and ranitidine (range: 26–95% decrease relative to non-ECMO controls) (9, 19, 39, 46). Increased clearance was reported for caspofungin, micafungin, clonidine, midazolam, morphine, and sildenafil (range: 25–455% increase relative to non-ECMO controls) (32, 34, 40, 43, 45, 47). Changes in clearance were not observed for cefotaxime, meropenem, vancomycin, oseltamivir, fluconazole, voriconazole, and levetiracetam (8, 15, 22, 27, 30, 34, 36).

Similarly, for vancomycin, the study by Buck (22), which included a non-ECMO comparator group within the same study, did not report a statistically significant difference in clearance. However, comparison of vancomycin clearance values in pediatric patients supported on ECMO against historical controls showed decreased clearance (20, 24, 25, 50). No significant association was found between log *P*-values of drugs and changes in clearance attributable to ECMO (Supplementary Table 1).

### Quality of Reporting of Published Pharmacokinetic Studies of Drugs in Pediatric Patients Supported on ECMO

Of the 24 items in the ClinPK checklist, all included studies reported the drugs and patient population studied. Majority of studies (>50%) also provided an explanation of the study rationale, objectives and hypotheses, eligibility criteria of study participants, information on ethics approval, drug preparation and administration techniques, details on blood sampling including quantification of drugs and their metabolites using validated approaches, pharmacokinetic modeling methods, and formulas for calculation of variables. The detailed percentages and lists of studies are tabulated in Supplementary Table 2. Majority of studies (>50%) also provided information on relevant variables that may account for inter- and intra-patient variability in pharmacokinetics, and pharmacokinetic parameters were reported appropriately for most studies (Supplementary Table 2). However, only 14 of the 41 included studies (34.1%) provided background information on the pharmacokinetic data that are known and relevant to the drugs being evaluated. In addition, only 16 of the 41 studies (39.0%) reported on the concurrent administration (or lack thereof) of study drugs with other drugs or food substances that may potentially interact with the study drugs. Study withdrawals or subjects lost to follow-up (or lack thereof), as well as quantification of missing or excluded data, were also rarely reported in only 10 and 2 studies (24.4 and 4.9%), respectively.

## Discussion

To our best knowledge, this is the first systematic review that encompasses drugs across all therapeutic classes and focuses on the available data in critically ill pediatric patients. Raffaeli et al. (70) recently provided an overview of the available evidence pertaining to determinants of altered drug disposition and evidence-based pharmacotherapy recommendations during ECMO specifically in neonates. The present study includes the available data in critically ill pediatric patients across different age groups. Using a comprehensive systematic search strategy, we identified a total of 41 studies evaluating 23 drugs. In line with the results of *ex vivo* studies that demonstrated drug extraction by the ECMO circuit, particularly for highly lipophilic and protein-bound drugs (2, 3) we found substantial pharmacokinetic alterations, in either drug clearance or *V*_*d*_ or both, reported in 70% of drugs studied in pediatric patients supported on ECMO.

Most studies demonstrated increased *V*_*d*_ and decreased clearance of drugs between ECMO and non-ECMO patients. The differences in pharmacokinetics for which we have the most confidence are those generated from studies that included the non-ECMO comparator groups. However, majority of the studies did not include the non-ECMO comparator groups, and the comparisons were made based on pharmacokinetic parameters reported in different studies. The differences in *V*_*d*_ and clearance of some of the studied drugs, such as vancomycin, between ECMO and non-ECMO controls showed marked intra-study variability, with some studies demonstrating increased values for the pharmacokinetic parameters (20, 21, 23, 24, 50) while others demonstrated decreased values or no change (22).

Despite our best efforts to compare these values across studies with similar patient demographics, it is unclear whether the differences, or lack thereof, are confounded by differences in patient profiles, study designs, and methodology. Notably, the lack of controlled data and inclusion of non-ECMO controls within these studies may have contributed to such heterogeneity. For this reason, it is difficult to draw definite conclusions regarding the pharmacokinetic differences between the ECMO and non-ECMO groups and further highlights the need for future studies evaluating the impact of ECMO on drug pharmacokinetics and disposition to include the non-ECMO comparator groups. Additionally, in contrast to the findings of *ex vivo* studies which showed the sequestration of drugs by the ECMO circuit to be particularly profound for more lipophilic drugs, we did not observe any correlation between log *P*-values of drugs and changes in both clearance and *V*_*d*_ attributable to ECMO. However, this may be due in part to the limited number of drugs studied to date, which makes it difficult to draw conclusive results.

There are several limitations to our systematic review. Firstly, most of the included studies had small sample sizes and were performed mainly in neonates and infants, with some studies including a mixed pediatric population. Given that the effect of ECMO on drug pharmacokinetics and disposition can vary by patient age, the extrapolation of these results to older children supported on ECMO cannot be robustly justified and requires dedicated pharmacokinetic trials to address. Importantly, the ECMO setup has evolved considerably over time. Considering that most of the studies were conducted in the 1990s, the differences in drug extraction between the older ECMO components and contemporary ECMO setup remain unclear and require further investigation.

The heterogeneity across studies, in terms of not only drugs studied but also clinical indications for which those drugs were administered and other patient factors, contributes substantially to large pharmacokinetic variability and may limit the conclusions that can be drawn. Such heterogeneity is further compounded by physiological derangements associated with critical illnesses, renal replacement therapy, drug–drug interactions, genetic polymorphisms, and the use of ECMO (71–74). Clearances of drugs in patients supported on ECMO are also determined by renal and hepatic flow and function. Unfortunately, these pieces of information were not included in most publications included in the review, and we were unable to address this in our analysis. A much deeper understanding of the interplay between these factors is critical in improving our ability to provide personalized dosing to pediatric patients on ECMO.

Pediatric pharmacokinetic research presents specific challenges, some of which can be circumvented with the use of model-based approaches to study design and analysis, such as population pharmacokinetic modeling and physiologically based pharmacokinetic modeling. As such, identification of the most optimal study design and pharmacokinetic protocol would be crucial for future pharmacology studies in pediatric ECMO patients. Furthermore, the transparent and complete reporting of study data in clinical pharmacokinetic studies is essential for better assessment and evaluation of study information and its clinical translation. Although validated tools for assessment of the quality and validity of pharmacokinetic studies have not yet been developed, the use of the ClinPK consensus in this systematic review has highlighted the poor compliance of most studies to the 24-item checklist considered to be necessary for the reporting of pharmacokinetic studies. We recommend more complete reporting of future pharmacokinetic studies that meet at least the minimum reporting criteria in this patient population. This may improve the utility and comparability of study findings and further circumvent the unique challenges associated with pediatric pharmacology studies.

In addition, the majority of pharmacokinetic data in ECMO-supported pediatric populations to date have been from studies conducted in neonates and infants, with antimicrobial agents and anticonvulsants being the most commonly studied drugs. Notably, studies evaluating pharmacokinetic changes of many drugs such as dexmedetomidine as well as other drug classes, including analgesics and cardiovascular, sedative, and anesthetic agents, which are commonly used in critically ill pediatric patients on ECMO, are still lacking and represent important areas of future studies. This highlights the urgent need for pharmacokinetic studies in these children for specific and clinically important drug classes, using a contemporary ECMO setup and with appropriate study designs, including the inclusion of appropriate controls.

## Conclusions

While the total number of drugs studied to date remains limited, we found substantial pharmacokinetic alterations in terms of *V*_*d*_ and/or clearance in 69.5% of drugs studied in children on ECMO. We also identified major limitations of the existing evidence base, which explains at least partially our current inability to readily predict pharmacokinetic changes and thus dose adjustments of drugs in critically ill children on ECMO. Systematic evaluations of pharmacokinetic alterations of drugs on ECMO that incorporate multidrug opportunistic trials, physiologically based pharmacokinetic modeling, *ex vivo* studies, and other methods are necessary for definitive dose recommendations.

## Data Availability Statement

All datasets generated for this study are included in the article/Supplementary Materials.

## Author's Note

This manuscript has been released as a preprint at Research Square, Sutiman et al. (75).

## Author Contributions

NS conceptualized and designed the study, obtained funding, designed the data collection instruments, collected data, carried out the analysis and interpretation of the data, and drafted and revised the manuscript. JL conceptualized and designed the study, obtained funding, collected data, carried out the analysis and interpretation of the data, and reviewed and revised the manuscript. JK collected data, carried out analysis, and interpretation of data and reviewed the manuscript. KW and CH collected data, carried out the analysis and interpretation of the data, and drafted, reviewed, and revised the manuscript. YC collected data, carried out the analysis and interpretation of the data, and reviewed and revised the manuscript. BM provided technical support, collected data, and carried out the analysis and interpretation of the data. All authors approved the final manuscript as submitted and agree to be accountable for all aspects of the work.

## Conflict of Interest

The authors declare that the research was conducted in the absence of any commercial or financial relationships that could be construed as a potential conflict of interest.
