# Supplementary material for: Pharmacokinetics Alterations in Critically Ill Pediatric Patients on Extracorporeal Membrane Oxygenation: A Systematic Review
Source: Front Pediatr. 2020 Jun 26;8:260. doi: 10.3389/fped.2020.00260 (PMC7332755; doi:10.3389/fped.2020.00260)
Supplement: Supplementary file 1 [file Data_Sheet_1.docx]

Supplementary Material

## Supplementary Table 1. Log *P* values of the studied drugs (obtained from PubChem, DrugBank and Human Metabolome Database). Positive log *P* values correlates with lipophilicity. Negative log *P* values correlates with hydrophilicity.

| Drug | Log *P* |
| --- | --- |
| Antibiotics |  |
| Cefotaxime | -0.5 |
| Gentamicin | -3.1 |
| Meropenem | -0.6 |
| Piperacillin | 0.3 |
| Tazobactam | -1.4 |
| Ticarcillin | 0.6 |
| Clavulanate | -1.5 |
| Vancomycin | -3.1 |
| Anti-virals |  |
| Acyclovir | -1.56 |
| Oseltamivir | 1 |
| Ribavirin | -1.85 |
| Anti-fungals |  |
| Caspofungin | 0 |
| Fluconazole | 0.5 |
| Micafungin | -1.5 |
| Voriconazole | 1 |
| Anti-convulsants |  |
| Fos-phenytoin | Unreported |
| Levetiracetam | -0.6 |
| Phenobarbital | 1.47 |
| Others |  |
| Bumetanide | 2.6 |
| Clonidine | 1.59 |
| Heparin | -13.2 |
| Midazolam | 3.33 |
| Morphine | 0.89 |
| Ranitidine | 0.2 |
| Sildenafil | 1.9 |

**Supplementary Table 2.** Quality of reporting of published pharmacokinetic studies of drugs in pediatric patients supported on ECMO based on the ClinPK checklist (adapted from Kanji S, Hayes M, Ling A, et al. Reporting Guidelines for Clinical Pharmacokinetic Studies: The ClinPK Statement. Clin Pharmacokinet. 2015. DOI 10.1007/s40262-015-0236-8.)

| ClinPK checklist items | No. of studies (%) | Refs |
| --- | --- | --- |
| 1. The title identifies the drug(s) and patient population(s) studied | 41 (100%) | 9-25, 27-47, 49-51 |
| 2. The abstract includes the name of the drug(s) studied, the route of administration, the population in whom it was studied, and the results of the primary objective and major clinical pharmacokinetic findings. | 39 (95.1%) | 9, 11-25, 27-44, 46, 47, 49-51 |
| 3. Pharmacokinetic data (i.e., absorption, distribution, metabolism, excretion) that is known and relevant to the drugs being studied is described | 14 (34.1%) | 10-12, 14, 20, 22, 27, 29, 30, 32, 39, 41, 46, 49 |
| 4. An explanation of the study rationale is provided | 39 (95.1%) | 9-25, 27-33, 35, 37-47, 49-51 |
| 5. Specific objectives or hypotheses is provided | 39 (95.1%) | 10-25, 27-37, 39-47, 49-51 |
| 6. Eligibility criteria of study participants is described | 27 (65.9%) | 11-17, 20-25, 27, 29-34, 40-44, 49, 51 |
| 7. Information about ethical approval of the study and subjects’ consent is provided. | 25 (61.0%) | 11-17, 20-23, 25, 27, 29-33, 41-44, 49-51 |
| 8. Co-administration (or lack thereof) of study drug(s) with other potentially interacting drugs or food within this study is described. | 16 (39.0%) | 10, 11, 19-21, 25, 27, 28, 34, 35, 41, 44-47, 50 |
| 9. Drug preparation and administration characteristics including dose, route, formulation, infusion duration (if applicable) and frequency are described. | 37 (90.2%) | 9-25, 27-33, 35, 39-47, 49-51 |
| 10. Body fluid or tissue sampling (timing, frequency and storage) for quantitative drug measurement is described. | 37 (90.2%) | 9-18, 20-25, 27, 29-35, 38-47, 49-51 |
| 11. Validation of quantitative bioanalytical methods used in the study is described in detail or described briefly and referenced. | 29 (70.7%) | 9-11, 13-16, 20-25, 27, 29-33, 35, 39-42, 44, 47, 49-51 |
| 12. Pharmacokinetic modeling methods, observed and derived parameters along with the formulas, and software used are described. | 36 (87.8%) | 9-11, 13,18, 20-25, 27, 29-35, 37-45, 57, 49-51 |
| 13. Formulas for calculated variables (such as creatinine clearance, body surface area, AUC, and adjusted body weight) are provided or referenced. | 21 (51.2%) | 9, 11, 15, 16, 20-22, 25, 27, 29, 31-33, 35, 41-44, 47, 50, 51 |
| 14. The specific body weight used in drug dosing and pharmacokinetic calculations are reported (i.e., ideal body weight vs. actual body weight vs. adjusted body weight) | 8 (19.5%) | 9, 11, 15, 19, 20, 22, 29, 34 |
| 15. Statistical methods including software used are described | 26 (63.4%) | 11, 12, 14-16, 20-22, 24, 25, 27, 29-34, 39-44, 47, 49, 51 |
| 16. Study withdrawals or subjects lost-to-follow up (or lack thereof) are reported. | 10 (24.4%) | 21-23, 25, 31, 33, 43, 44, 49, 51 |
| 17. Quantification of missing or excluded data is provided if applicable. | 2 (4.9%) | 21, 23 |
| 18. All relevant variables that may explain inter- and intra-patient pharmacokinetic variability (including: age, sex, end-organ function, ethnicity, weight or BMI, health status or severity of illness, and pertinent co-morbidities) are provided with appropriate measures of variability. | 34 (82.9%) | 9-17, 19-22, 24, 25, 27-33, 35, 36, 39, 40, 42-47, 49-51 |
| 19. Results of pharmacokinetic analyses are reported with appropriate measures of variability and precision (such as range, standard deviation, 95% confidence interval, etc.) | 40 (97.6%) | 10-25, 27-47, 49-51 |
| 20. Studies in patients receiving extracorporeal drug removal (i.e., dialysis) should report the mode of drug removal, type of filters used, duration of therapy and relevant flow rates. | 30 (73.7%) | 9, 11-16, 20-25, 2735, 39, 41, 42, 44, 46, 47, 50, 51 |
| 21. In studies of drug bioavailability comparing two formulations of the same drug, F (bioavailability), AUC, C_max_ (maximum concentration) and t_max_ (time to maximum concentration) should be reported. | 0 (0%) | - |
| 22. Study limitations describing potential sources of bias and imprecision where relevant should be described | 14 (34.1%) | 11, 12, 16, 17, 21, 23, 25, 27, 29, 30, 39, 42, 43, 51 |
| 23. The relevance of study findings (applicability, external validity) is described | 36 (87.8%) | 8-17, 20-25, 27-33, 35, 38-47, 49-51 |
| 24. Funding sources and conflicts of interest for the authors are disclosed. | 15 (36.6%) | 12, 15-17, 20, 22, 23, 25, 27, 39, 42, 45-47, 50 |

**Supplementary Information 1.** Detailed search strategies

**MEDLINE**

((((("extracorporeal membrane oxygenation"[MeSH Terms] OR "extracorporeal membrane oxygenation"[tw] OR "ecmo"[tw])) AND (((((("pharmacokinetics"[Subheading] OR "pharmacokinetics"[tw] OR "pharmacokinetic"[tw] OR "pharmacokinetics"[MeSH Terms])) OR ((drug[tw] AND disposition[tw]) OR (drugs[tw] AND disposition[tw]))) OR ("pharmacology"[Subheading] OR "pharmacology"[tw] OR "pharmacology"[MeSH Terms])) OR (dosing [tw] OR doses [tw] OR dose [tw] OR dosed [tw] OR dosage [tw] OR dosages [tw])) OR ("administration and dosage" [Subheading]))) AND ((infant[MeSH] OR child[MeSH] OR adolescent[MeSH])))) OR ((((("extracorporeal membrane oxygenation"[MeSH Terms] OR "extracorporeal membrane oxygenation"[tw] OR "ecmo"[tw])) AND (((((("pharmacokinetics"[Subheading] OR "pharmacokinetics"[tw] OR "pharmacokinetic"[tw] OR "pharmacokinetics"[MeSH Terms])) OR ((drug[tw] AND disposition[tw]) OR (drugs[tw] AND disposition[tw]))) OR ("pharmacology"[Subheading] OR "pharmacology"[tw] OR "pharmacology"[MeSH Terms])) OR (dosing [tw] OR doses [tw] OR dose [tw] OR dosed [tw] OR dosage [tw] OR dosages [tw])) OR ("administration and dosage" [Subheading])))) AND (pediatric [tw] OR pediatrics [tw] OR paediatric [tw] OR paediatrics [tw] OR newborn [tw] OR newborns [tw] OR neonate [tw] OR neonates [tw] OR neonatal [tw] OR infant [tw] OR infants [tw] OR infancy [tw] OR child [tw] OR children [tw] OR adolecent [tw] OR adolecents [tw] OR adolescense [tw] OR adolescence [tw] OR adolescent [tw] OR adolescents [tw] OR teen [tw] OR teenager [tw] OR teenagers [tw])) Filters: English

**CINAHL**

((ecmo OR "extracorp* membran* oxygenat*"") OR (MH "Extracorporeal Membrane Oxygenation")) AND ((administration and dosage) OR MW "pk" OR pharmacokinetic* OR (MH "Pharmacokinetics+") OR pharmacolog* OR (MH "Pharmacy and Pharmacology") OR (drug* AND disposition) OR MW "ad" OR (dosing OR dose* OR dosag*)) AND [limit to Age Groups: Fetus-18 years] OR [combine terms with] ((pediatric* OR paediatric* OR newborn* OR neonat* OR infant* OR infancy OR child* OR adolesc* OR adolecen* OR teen*))

**Embase**

(('extracorporeal oxygenation'/exp OR 'extracorporeal oxygenation' OR 'extracorporeal membrane oxygenation'/exp OR 'extracorporeal membrane oxygenat*' OR 'ecmo') AND ('administration and dosage' OR 'drug dose' OR 'dosing' OR 'dose*' OR 'dosag*' OR 'pharmacology' OR 'pharmacokinetic assay' OR 'pharmacokinetic*' OR 'drug disposition*') AND ([adolescent]/lim OR [child]/lim OR [embryo]/lim OR [fetus]/lim OR [infant]/lim OR [newborn]/lim OR [preschool]/lim OR [school]/lim) OR (('extracorporeal oxygenation'/exp OR 'extracorporeal oxygenation' OR 'extracorporeal membrane oxygenation'/exp OR 'extracorporeal membrane oxygenat*' OR 'ecmo') AND ('administration and dosage' OR 'drug dose' OR 'dosing' OR 'dose*' OR 'dosag*' OR 'pharmacology' OR 'pharmacokinetic assay' OR 'pharmacokinetic*' OR 'drug disposition*') AND ('pediatric*' OR 'paediatric*' OR 'newborn*' OR 'neonat*' OR 'infant*' OR 'infancy' OR 'child*' OR 'adolesc*' OR 'adolecen*' OR 'teen*'))) AND [english]/lim
